# Akutversorgung psychisch kranker Kinder und Jugendlicher: Eine Pilotstudie an 257 PatientInnen

**DOI:** 10.1007/s40211-021-00389-7

**Published:** 2021-03-15

**Authors:** Teresa Eichinger, Elisabeth Marte, Leonhard Thun-Hohenstein, Franz Santner, Belinda Plattner

**Affiliations:** 1 Kepler Universitätsklinikum, Linz, Österreich; 2 Universitätsklinik für Kinder- und Jugendpsychiatrie, Salzburger Landeskliniken (SALK) – Universitätsklinikum der Paracelsus Medizinischen Privatuniversität, Ignaz Harrerstraße 79, 5020 Salzburg, Österreich

**Keywords:** Psychiatrischer Notfall, Geschlecht, Akutbehandlung, Suizidalität, Aggression, Psychiatric emergency, Gender, Acute treatment, Suicidality, Aggression

## Abstract

**Fragestellung:**

Nicht bewältigte Entwicklungsaufgaben, schwierige soziale Rahmenbedingungen, aber auch psychische Erkrankungen können in Krisen münden, die zu einer Akutvorstellung an der Kinder- und Jugendpsychiatrie (KJP) führen. Ziel der vorliegenden Studie war es eine repräsentative Inanspruchnahmepopulation akut vorstelliger Patienten zu untersuchen, um einen Überblick über die Nutzung des akutpsychiatrischen Angebotes in der Kinder- und Jugendpsychiatrie zu erhalten.

**Methodik:**

Es handelt sich um eine retrospektive Analyse der aus dem Ersteinschätzungsblatt und aus dem Krankenhaus internen Datenverarbeitungssystem erhobenen Daten. Untersucht wurden akut vorstellige Kinder und Jugendliche an Salzburgs einzigem Akutspital mit Unterbringungsbereich für diese Altersgruppe.

**Ergebnisse:**

Von den 257 vorstelligen Patienten waren 53,7 % weiblich. Signifikant häufiger werden Jungen wegen externalisierender Symptome oder Außenfaktoren, Mädchen hingegen wegen suizidaler Symptome vorstellig. Zumeist bestehen die Symptome länger als eine Woche, bei einem Teil der Patienten sogar länger als 6 Monate. 51 % der Kinder und Jugendlichen wurden stationär aufgenommen, 43,2 % aller Vorgestellten nach Unterbringungsgesetz. Im Unterbringungsbereich betrug die Aufenthaltsdauer im Mittel drei Nächte.

**Schlussfolgerung:**

Viele Kinder und Jugendliche zeigen bei Akutvorstellung eine chronifizierte Symptomatik. Suizidalität war ein häufiger Vorstellungsgrund, weshalb eine Unterbringung oftmals indiziert war. Die Erarbeitung von Akutkonzepten, insbesondere primäre, sekundäre und tertiäre Suizidprävention aber auch der Ausbau von niederschwelligen Anlaufstellen zeigen sich als wesentliche Zukunftsherausforderungen für die Kinder- und Jugendpsychiatrie in Österreich.

**Zusatzmaterial online:**

Zusätzliche Informationen sind in der Online-Version dieses Artikels (10.1007/s40211-021-00389-7) enthalten.

## Abstract (en)

**Objective:**

Challenging development tasks, problematic social environments and psychiatric disorder can result in crisis leading to an emergency consultation at child and adolescent psychiatry (CAP). The study aims to describe a representative clinical sample of patients seeking help at an acute CAP outpatient clinic.

**Methods:**

Data from the initial assessment tool for CAP and data gained from the hospital IT-System were analyzed. The study site is the only hospital in the country of Salzburg in Austria with a specialized emergency unit for CAP patients.

**Results:**

Out of the 257 patients, 53.7% were female. Boys significantly more often presented with externalizing symptoms and because of external factors. Girls significantly more often reported the presence of suicidal thoughts. Symptoms that led to consultation often were present for more than a week prior to consultation, in some cases even longer than 6 months. 51% of the children and adolescents were treated at the  inpatient unit, 43% at the closed unit, where they stayed for a mean of 3 nights.

**Conclusions:**

Many patients, who attended the child and adolescent psychiatry emergency showed a long duration of presenting symptoms. Suicidal tendency was a common reason for consultation and often led to a closed  inpatient treatment. Developing concepts for acute situations—like primary, secondary and tertiary suicide prevention—as well as improving the easier access to child and adolescent psychiatric services seems necessary.

## Einleitung

Die Kindheits- und Jugendjahre sind eine Zeit des körperlichen, sozialen und innerpsychischen Wandels. Entwicklungsaufgaben sind zu meistern, dafür ist die Adaption des Kindes/des Jugendlichen und seines Umfeldes an sich im ständigen Wechsel befindliche Herausforderungen notwendig. Diskrepanzen zwischen gesellschaftlich geforderten Entwicklungsschritten (z. B. Übertritt in höhere Schule) und dem Entwicklungsstand des Kindes, der Belastbarkeit der Familien oder der Betreuungssysteme und den individuellen Fähigkeiten des Kindes führen häufig zu relevanten Krisen [1]. Eine Krise im Kindes- und Jugendalter kann aber auch direkt im Zusammenhang mit einer psychiatrischen Erkrankung auftreten. Viele psychische Störungen manifestieren sich erstmals im Kindes- und Jugendalter [2]. Die Punktprävalenz psychischer Störungen bei Kindern und Jugendlichen wurde mit 24 % als hoch eingestuft [3].

Zweifelsohne besteht ein hoher Bedarf an kinder- und jugendpsychiatrischer Betreuung und Behandlung. Mit Bedauern muss festgestellt werden, dass in Österreich die kinder- und jugendpsychiatrische Versorgung noch nicht ausreichend ausgebaut ist. Im Österreichischen Strukturplan Gesundheit (ÖSG) wurde als Zielgröße eine Bettenmessziffer von 0,08 bis 0,13 pro 1000 Einwohner festgesetzt. Trotz rezenter Verbesserungen im stationäre Ausbau liegt mit Stand 2018 die Bettenmessziffer unter dem geforderten Wert von 0,1 ‰ [4]. Ein niedriger Bettenschlüssel kann zu Engpässen in der stationären Versorgung akut behandlungsbedürftiger Kinder und Jugendlicher führen.

Im extramuralen Bereich – dies fasst die Behandlung durch die niedergelassenen Fachärzte und durch Ambulatorien zusammen – ist ein noch größerer Mangel spürbar. In Österreich sind mit Stand August 2017 nur ein Viertel der benötigten Kassenstellen besetzt – in manchen Bundesländern (Steiermark und Burgenland) bestehen keine Kassenverträge für Kinder- und Jugendpsychiatrie [4]. In Salzburg sind zwei von sieben geplanten, niedergelassenen Facharztstellen verfügbar. In strukturell besser ausgebauten Regionen erfolgt die Versorgung von Kindern und Jugendlichen mit psychischen Krisen und Erkrankungen auch durch Psychotherapeuten und Kinderpsychologen, jedoch in ländlichen Regionen zum Teil durch den Haus- oder Kinderarzt. Bei geringen extramuralen Kapazitäten kann nur ein sehr geringer Anteil der behandlungsbedürftigen Kinder und Jugendlichen zeitnahe adäquat versorgt werden. Dies könnte bewirken, dass durch einen verspäteten Behandlungsbeginn Krisen dementsprechend akuter werden und dadurch Patienten eine stationäre Behandlung benötigen.

Demnach stellen sich die Fragen, in welcher Form mit psychischen Krisen im Kindes- und Jugendalter umgegangen werden soll, welcher Bedarf an Akutversorgung notwendig ist und wie sich die Kinder- und Jugendpsychiatrie in den kommenden Jahrzehnten entwickeln soll. Während in der Erwachsenenpsychiatrie bereits fundierte Konzepte und Vorgehensweisen etabliert sind, steht unser junges Fach vor einer Herausforderung.

Prognostisch wichtig erscheint hierbei eine gezielte und fachlich richtige Entscheidung bei der Akutvorstellung an der Kinder- und Jugendpsychiatrie. Hier gilt es Entwicklungskrisen nicht zu pathologisieren und empfohlene Hilfestellungen und Empfehlungen den Umständen anzupassen. Andererseits müssen schwere psychiatrische Erkrankungen (bipolare Störung, Schizophrenie) erkannt und sofort behandelt werden. Ferner sollte das Risiko für Selbst- und Fremdgefährdung erfasst und damit der Schutz des Kindes/Jugendlichen und der Gesellschaft gewährleistet werden [5].

## Methodik

Die Stichprobe umfasst alle Kinder und Jugendlichen (4.–23. Lebensjahr), die von Juni 2016 bis Juli 2017 in der Akutambulanz der Universitätsklinik für Kinder- und Jugendpsychiatrie an den Salzburger Landesklinken vorstellig waren. Die Klinik hat akute Versorgungspflicht für das Bundesland Salzburg (550.000 Einwohner) und hat als einzige stationäre Einrichtung im Bundesland die Möglichkeit zur Unterbringung von Kindern und Jugendlichen nach dem Unterbringungsgesetz [6]. Das maximale Behandlungsalter liegt bei 23 Jahren, da zum Studienzeitpunkt im Bundesland Salzburg eine Vereinbarung bestand, dass Patienten mit Essstörungen bis zum 24. Lebensjahr an der Kinder- und Jugendpsychiatrie versorgt werden. Die Auswertung und Publikation der Daten wurde von der Ethikkommission des Bundeslandes Salzburg bewilligt (415-E/2231/4-2017).

Die Daten wurden mithilfe eines für die Ersteinschätzung akut vorstelliger Patienten entworfenen Instrumentes erhoben. Das Ersteinschätzungsblatt wurde im Rahmen des Qualitätsmanagementprozesses durch Oberärzte und dem Leiter der Pflege entworfen und ermöglicht die Erhebung folgender Themen: Fragen zur Person: Vorstellungsgrund, Pflege‑/Arztkontakt im Akutsetting mit Zeitangaben, Vorliegen einer konsiliarischen Zuweisung und bereits vorbestehende Kontakte zur Universitätsklinik für Kinder- und Jugendpsychiatrie, Fragen zur Akutsituation: Die Fragen betreffen das Vorliegen einer Einweisung nach Paragraph 8 oder nach Paragraph 9 des österreichischen Unterbringungsgesetzes, die Unfähigkeit altersadäquat auf Ansprache zu reagieren, das Vorhandensein von Suizidgedanken, das Bestehen fremdgefährlichen Verhaltens, die Unfähigkeit einer altersadäquaten Orientierung zur Person/Ort/Situation, das Vorhandensein starker Unruhe/Erregung und die Fluchtgefahr, Dauer der Symptomatik, Vitalparameter, drei standardisierte Skalen: Schmerzskala, Suizidskala und Aggressionsskala und ein freies Textfeld mit der Möglichkeit für relevante Notizen.

Das Ersterhebungsblatt ist in der Beilage angefügt. Das Instrument wurde von der bei Akutvorstellung zuständigen Pflegeperson angewandt, bevor der Dienstarzt kontaktiert wurde. Ferner konnten wir auf Daten aus dem Krankenhaus internen Datenverarbeitungssystemes (ORBIS) zugreifen.

## Statistik

Die Analyse der Daten erfolgte mit dem Statistical Package for Social Sciences Version 24 (SPSS Inc, Chicago, IL). Es wurden deskriptive Statistik, Häufigkeiten und Mittelwerte mit Standardabweichung, Korrelationskoeffizienten, Chi-Quadrat-Tests, Kreuztabellen und T‑Tests angewandt.

## Ergebnisse

Innerhalb des Studienzeitraumes (14 Monate) gab es 382 Akutvorstellungen von insgesamt 257 vorstelligen Patienten. Davon waren 46,3 % (*n* = 119) männlich und 53,7 % (*n* = 138) weiblich. Das Durchschnittsalter lag bei 14,7 Jahren (SD = 2,53). Es gibt keine signifikanten geschlechtsspezifischen Unterschiede beim Alter der kinder- und jugendpsychiatrischen Patienten in der Akutambulanz (t (254) = 0,230, *p* = 0,469, zweiseitig).

### Konsil und Bekanntheitsgrad an der Klinik

30,2 % der Akutpatienten (*n* = 76 von *n* = 252) wurden mit konsiliarischer Anfrage von anderen ärztlichen Kollegen vorgestellt. 28,4 % (*n* = 73 von *n* = 257) der vorstelligen Patienten waren bereits an der Abteilung bekannt. Diese 73 Personen sind im Mittel 20,79 Monate (SD = 19,83) bekannt, mit einem Minimum von 0 Monaten (6,8 %, *n* = 5) und einem Maximum von 79 Monaten (1,4 %, *n* = 1). 43,8 % (*n* = 32) der Kinder und Jugendlichen sind unter 12 Monate, 20,5 % (*n* = 15) unter 24 Monate, 17,8 % (*n* = 13) unter 36 Monate und 17,8 % (*n* = 13) über 36 Monate bekannt.

### Vorstellungsgrund

Die Vorstellungsgründe wurden offen nach Angabe des Patienten vermerkt. Nennungen von depressiver Stimmung, körperlichen Beschwerden, Angst und Essstörung wurden unter internalisierende Symptome; psychotische Symptome, Substanzmittelabusus und aggressives Verhalten wurde unter externalisierende Symptome; Suizidalität, Selbstverletzung und Suizidversuche wurden unter suizidale Symptome und Probleme in der Familie oder in Schule/Arbeit wurden unter Außenfaktoren zusammengefasst. Die genannten Vorstellungsgründe in der Akutambulanz bei den männlichen und weiblichen Kindern und Jugendlichen sind in der Tab. 1 dargestellt. Mit über 50 % war der häufigste akute Vorstellungsgrund Suizidalität, wobei signifikant mehr Mädchen als Jungen diese angaben. Signifikant häufiger werden Jungen wegen externalisierender Symptome und Außenfaktoren vorstellig.

**Table 1:** 

Vorstellungsgrund	Gesamt (*n* = 257)	Jungen (*n* = 119)	Mädchen (*n* = 138)	X^2^ (df)	*p*
Internal. Sympt	54 (21 %)	20 (16,8 %)	34 (24,6 %)	2,36 (1)	0,124
External. Sympt	65 (25,3 %)	37 (31,1 %)	28 (20,3 %)	3,95 (1)	*0,047*
Suizidale Sympt	145 (56,4 %)	58 (48,7 %)	87 (63 %)	5,32 (1)	*0,021*
Außenfaktoren	32 (12,5 %)	21 (17,6 %)	11 (7,9 %)	5,49 (1)	*0,019*
Sonstiges	14 (5,4 %)	7 (5,8 %)	7 (5 %)	0,08 (1)	0,775

### Fragen zu kritischen Parametern in psychiatrischen Akutsituationen

In der Tab. 2 sind die Prävalenzen der mittels Instrument erhobenen klinischen Parameter für die Einschätzung der akuten Situation dargestellt. Suizidgedanken waren mit über 50 % der häufigste klinische Akutparameter, gefolgt von klinisch beobachtbarer Unruhe/Erregung. Signifikant häufiger als bei den Mädchen waren bei den Jungen die Einweisung nach Paragraph 8 oder 9 und eine Fremdgefährdung. Bei den Mädchen hingegen waren Suizidgedanken und eine beobachtete Unruhe/Erregung signifikant häufiger.

**Table 2:** 

Klinische Parameter	Gesamt (*n* = 257)	Jungen (*n* = 119)	Mädchen (*n* = 138)	X^2^(df)	*p*
§ 8 od 9 UBG	47 (18,4 %)	33 (28 %)	14 (10,1 %)	13,478 (1)	*0,000*
Inadäqu. Reakt	21 (8,4 %)	11 (9,6 %)	10 (7,4 %)	0,398 (1)	0,528
Suizidgedanken	135 (53,8 %)	48 (41,7 %)	87 (64 %)	12,390 (1)	*0,000*
Fremdgefährdung	19 (7,5 %)	13 (11,3 %)	6 (4,4 %)	4,301 (1)	*0,038*
Nicht orientiert	21 (8,4 %)	10 (8,6 %)	11 (8,2 %)	0,014 (1)	0,907
Unruhe/Erregung	77 (30,7 %)	28 (24,3 %)	49 (36 %)	3,998 (1)	*0,046*
Fluchtgefahr	14 (5,5 %)	8 (6,8 %)	6 (4,4 %)	0,732 (1)	0,392

### Dauer der Symptomatik

In Tab. 3 wird angegeben, wie lange die zur Vorstellung führenden Symptome in ähnlicher Art/Intensität bereits vorhanden waren. Auffallend ist, dass bei 60 % der Jungen die Symptome länger als 1 Woche und bei wiederum 20 % länger als 6 Monate bestanden. Bei den Mädchen bestanden bei 73,5 % die Symptome länger als eine Woche und wiederum bei 29,9 % länger als 6 Monate. Signifikant häufiger als die Mädchen stellten sich die Jungen mit Symptomen unter 24 h Symptomdauer vor.

**Table 3:** 

Symptome	Gesamt (*n* = 257)	Jungen (*n* = 119)	Mädchen (*n* = 138)	X^2^(df)	*p*
< 24 h	22 (14,5 %)	16 (24,6 %)	6 (6,9 %)	9,436 (1)	*0,002*
24 h bis < 1 W	27 (17,8 %)	10 (15,4 %)	17 (19,5 %)	0,440 (1)	0,507
1 W < 1 M	36 (23,7 %)	13 (20 %)	23 (26,4 %)	0,853 (1)	0,356
1 M < 6 M	28 (18,4 %)	13 (20 %)	15 (17,2 %)	0,188 (1)	0,664
> 6 Monate	39 (25,7 %)	13 (20 %)	26 (29,9 %)	1,906 (1)	0,167

### Selbsteinschätzung von Suizidalität

In Tab. 4 ist die dokumentierte Einschätzung der Suizidalität durch die Patienten selbst nach der Suizidskala bei männlichen und weiblichen Kindern und Jugendlichen dargestellt. Hier gaben 75,3 % der Jungen und 58,3 % der Mädchen keine, kaum oder mäßige Suizidgedanken an, während 24,8 % der Jungen und 41,6 % der Mädchen drängende, sehr drängende Suizidgedanken oder eine suizidale Einengung anmerkten. Signifikant häufiger als Mädchen stellten sich Jungen mit keiner vor. Wohingegen Mädchen signifikant häufiger als Jungen über sehr drängende Suizidalität berichteten.

**Table 4:** 

Suizidalität	Gesamtstichprobe (*N* = 241)	Männliche Jugendliche (*N* = 109)	Weibliche Jugendliche (*N* = 138)	Wert (df)	*p*
Keine	117 (48,5 %)	67 (61,5 %)	50 (37,9 %)	13,29 (1)	*0,000*
Kaum	14 (5,8 %)	5 (4,6 %)	9 (6,8 %)	0,54 (1)	0,461
Mässig	28 (11,6 %)	10 (9,2 %)	18 (13,6 %)	1,16 (1)	0,280
Drängend	45 (18,7 %)	17 (15,6 %)	28 (21,2 %)	1,24 (1)	0,265
Sehr drängend	30 (12,4 %)	7 (6,4 %)	23 (17,4 %)	6,63 (1)	*0,010*
Eingeengt	7 (2,9 %)	3 (2,8 %)	4 (3,0 %)	0,02 (1)	0,898

### Behandlungsprozedere

Die Akutvorstellung führte bei 51 % zu einer stationären Aufnahme (*n* = 131 von *n* = 257), bei den Jungen waren es 51,3 % (*n* = 61 von *n* = 119) und bei den Mädchen 50,7 % (*n* = 70 von *n* = 138) der Akutvorstelligen.

Eine Behandlung im Unterbringungsbereich fand bei 43,2 % (*n* = 111 von *n* = 257) statt, bei den weiblichen bei 41,3 % (*n* = 57 von *n* = 138) und bei den männlichen bei 45,4 % (*n* = 54 von *n* = 119). Die Häufigkeit der Unterbringung zeigt keinen geschlechtsspezifischen signifikanten Unterschied (X^2^(1) = 0,432, *p* > 0,05 (zweiseitig)).

Es zeigte sich ein signifikanter Zusammenhang sowohl bezüglich der Selbsteinschätzung der Suizidalität durch die Patienten als auch der Fremdbeurteilung der kritischen Parameter psychiatrischer Akutsituationen durch die Pflege in Hinblick auf das Behandlungsprozedere. Die stationär aufgenommenen Patienten gaben eine drängendere Suizidalität an und deutlich mehr Fragen zu kritischen Parametern psychiatrischer Akutsituationen wurden durch das Pflegepersonal mit „Ja“ beantwortet. Dies zeigte sich bei den Aufnahmen auf der Normalstation (t(227) = −7,519, *p* = 0,000) und im Unterbringungsbereich (t(227) = −6,484, *p* = 0,000).

### Klinikaufenthalt in Nächten

Die 42 Jugendlichen, die nach der Akutvorstellung im offenen Bereich aufgenommen waren (entweder direkt offene Aufnahme oder nach Unterbringung), blieben im Mittel 19,62 Nächte aufgenommen (Minimum = 1, Maximum = 78, SD = 21,997), Buben im Mittel 17,1 Nächte (SD = 23,530), Mädchen 21,04 Nächte (SD = 21,425).

Die Patienten, die gemäß UBG im geschlossenen Bereich aufgenommen waren, waren im Mittel 2,74 Nächte (*n* = 111; SD = 5,93) untergebracht, Buben im Mittel 2,81 Nächte (*n* = 54; SD = 7,75) und Mädchen im Mittel 2,67 Nächte (*n* = 57; SD = 3,50).

### Diagnose bei stationärer Aufnahme

Wie in Tab. 5 ersichtlich wurde bei den stationär aufgenommenen Mädchen signifikant häufiger als bei den Jungen eine Diagnose aus der Kategorie Affektive Störungen (F3) gestellt, während die Buben signifikant häufiger eine Diagnose aus F9 (Verhaltens und emotionale Störungen mit Beginn in der Kindheit und Jugend) erhielten. Insgesamt erhielten sowohl Mädchen als auch Buben am häufigsten eine Diagnose aus der Kategorie Neurotische, Belastungs- und somatoforme Störungen (F4).

**Table 5:** 

Diagnose	Station (*N* = 131)	Männliche Jugendliche (*N* = 61)	Weibliche Jugendliche (*N* = 70)	Wert (X^2^)	*P*
F0	1 (0,8 %)	1 (1,6 %)	0 (0 %)	1,156	0,282
F1	10 (7,6 %)	7 (11,5 %)	3 (4,3 %)	2,390	0,122
F2	2 (1,5 %)	0 (0 %)	2 (2,9 %)	1,770	0,183
F3	15 (11,5 %)	2 (3,3 %)	13 (18,6 %)	7,518	*0,006*
F4	76 (58,0 %)	37 (60,7 %)	39 (55,7 %)	0,327	0,568
F5	2 (1,5 %)	0 (0 %)	2 (2,9 %)	1,770	0,183
F6	9 (3,5 %)	2 (3,3 %)	7 (10,0 %)	2,301	0,129
F8	2 (1,5 %)	2 (3,3 %)	0 (0 %)	2,331	0,127
F9	14 (10,7 %)	10 (16,4 %)	4 (5,7 %)	3,895	*0,048*

Innerhalb der 76 Patienten, die eine Diagnose mit F4 erhielten, erhielten 19,7 % (*n* = 15) die Diagnose akute Belastungsreaktion, 22,4 % (*n* = 17) die Diagnose Posttraumatische Belastungsstörung, 56,6 % (*n* = 43) die Diagnose Anpassungsstörung und 1,3 % (*n* = 1) die Diagnose F43.8 sonstige Reaktion auf schwere Belastungen.

### Akute Wiedervorstellungen

Ausgehend von der ersten mittels Ersteinschätzungsblatt dokumentierten Vorstellung im Studienzeitraum wurden die Patienten bis zum Juli 2017 weiterverfolgt. Relevant für unsere Studie waren die Dauer bis zur ersten Wiedervorstellung in Tagen, die Weiterbehandlung (ambulant, stationär oder stationär im Unterbringungsbereich) und die Anzahl der weiteren akuten Vorstellungen in der Klinik.

Im Beobachtungszeitraum stellten sich 21,8 % (*n* = 56) der Gesamtstichprobe (*n* = 257) erneut akut in der Akutambulanz vor. Das sind 26,9 % (*n* = 32) der Jungen und 17,4 % (*n* = 24) der Mädchen. 65,5 % (*n* = 36 von *n* = 55) wurden stationär aufgenommen, wobei 61,3 % (*n* = 19) Jungen und 70,8 % (*n* = 17) Mädchen waren. 49,1 % (*n* = 27 von *n* = 55) wurden laut UBG untergebracht, dabei handelte es sich um 48,4 % (*n* = 15) männliche und 50,0 % (*n* = 12) weibliche Patienten.

Im Mittel stellten sich die Kinder und Jugendlichen nach 56,57 Tagen (*n* = 56; SD = 61,21) wieder vor. Das Maximum lag bei 322 Tagen (*n* = 1 von *n* = 56) und das Minimum bei 0 Tagen (*n* = 1 von *n* = 56). Bei den Jungen war der Mittelwert der Wiedervorstellungen 55,47 Tage (*n* = 32, SD = 55,42) und bei den Mädchen 58,04 Tage (*n* = 24, SD = 69,39).

Die Kinder und Jugendlichen, welche sich erneut in der Akutambulanz vorstellten, sind im Mittel 2,23 (*n* = 56; SD = 3,24) Mal wiedervorstellig gewesen.

### Psychiatrische Notfälle (Selbst‑/Fremdgefährdung, Aggression)

25,9 % der akut vorstelligen Patienten kamen aufgrund von Selbst- oder Fremdgefährdung in die Ambulanz. Es zeigte sich, dass die Jungen deutlich häufiger aufgrund eines derartigen psychiatrischen Notfalles vorstellig waren.

Fremdaggression war kaum der Fall. Nur bei 0,8 % (*n* = 2 von *n* = 241) wurde angegeben, dass die Gewalt als Mittel zur Zielerreichung verwendet wurde.

## Diskussion

Diese Publikation gibt einen Überblick über jene Kinder und Jugendlichen, die eine akute Versorgung in der Klinik für Kinder- und Jugendpsychiatrie der Salzburger Landeskliniken (SALK) im Zeitraum von 14 Monaten (Juni 2016 bis einschließlich Juli 2017) in Anspruch genommen haben.

Für über 70 % stellte die Akutvorstellung den Erstkontakt mit der Klinik dar und dies in einem Durchschnittsalter von 14,7 Jahren. In diesem Alter befinden sich die Jugendlichen gerade in der Adoleszenz, also im Übergang von der Kindheit ins Erwachsenenalter. Da dieser Prozess durch Veränderungen sowohl biologischer (Entwicklung der sekundären Geschlechtsmerkmale, hormonelle Veränderungen sowie Reifungsprozesse im Gehirn) als auch psychosozialer (Erwerbung der Identität) Natur geprägt ist, können bei den Herausforderungen der Entwicklungsphasen akute Störungen der Anpassung auftreten [1]. Dies spiegelt sich auch in der am häufigsten gestellten Diagnose „Anpassungsstörung“ wider.

Nebst der Zeit der Bewältigung von Entwicklungsaufgaben stellt die Adoleszenz die Zeit der Erstmanifestation von psychiatrischen Erkrankungen dar. 50 % der psychischen Erkrankungen im Erwachsenenalter beginnen im Jugendalter [7]. Depressionen treten ab dem 13. Lebensjahr deutlich häufiger auf und neigen bei bis zu 80 % zu einer Chronifizierung bzw. weisen hohe Rezidivraten auf [8], was im Erwachsenenalter in Form von häufigen Hospitalisierungen spürbar werden kann. Bei Psychosen ist dies ähnlich, dabei steigt die Erkrankungshäufigkeit ab dem 15. Lebensjahr steil an [9]. Unter 13 Jahren werden hingegen selten Psychosen beobachtet [10]. In der Analyse akut vorstelliger Kinder und Jugendlicher ist aufgefallen, dass Mädchen signifikant häufiger mit affektiven Störungen diagnostiziert werden, wohingegen Jungen häufiger wegen externalisierender Symptome und Außenfaktoren vorstellig wurden und im Verlauf häufiger mit Verhaltensstörungen diagnostiziert wurden.

Bei über 50 % der akuten Patienten stellte Suizidalität den Vorstellungsgrund dar. Hier ist zu berücksichtigen, dass bis zum 21. Lebensjahr der Suizid die zweithäufigste Todesursache nach dem Unfalltod ist und dies am häufigsten im Alter zwischen 15 und 20 Jahren [11]. Nur sehr selten kommt es zu Suiziden vor der Pubertät [12]. Jungen begehen fast doppelt so häufig Suizide wie Mädchen [13]. Dennoch gaben signifikant mehr Mädchen eine „sehr drängende Suizidalität“ an. In der Literatur wird dieses Phänomen als Gender Paradoxon bezeichnet. Darunter wird verstanden, dass charakteristischerweise Jungen vermehrt Suizide und Mädchen vermehrt nicht-tödliche Suizidversuche (sozusagen als „Hilferuf“) begehen [14, 15]. Die Aktualität der Akutsituation in unserer Stichprobe zeigt sich auch darin, dass viele Patienten nach Vorstellung in der Akutambulanz stationär versorgt werden mussten (50 % der Vorstelligen) und sogar über 40 % der Kinder und Jugendlichen im Unterbringungsbereich.

Zu dem Thema Suizidalität stellt sich die drängende Frage, wie eine Regression von Suiziden im Jugendalter erreicht werden kann. In der Literatur werden drei Arten der Suizidprävention beschrieben. Die primäre Prävention soll das Auftreten von Suizidalität verhindern, auslösenden Faktoren entgegen wirken [16, 17] und somit Risikofaktoren senken. Dies könnte beispielsweise durch Vorträge gelingen, welche das Bewusstsein für die hochrelevante Thematik „Suizidalität im Jugendalter“ erhöhen und Jugendlichen zeigen sollen, wann Hilfe gesucht werden soll und wo sie diese finden können. Ziel ist es eine Sensibilisierung der Gesellschaft zur Früherkennung von typischen Anzeichen von Suizidalität zu erreichen sowie den richtigen Umgang mit Betroffenen zu lehren [17]. Die sekundäre Prävention soll bei der Früherkennung und Behandlung von Suizidalen helfen [16] sowie Betroffene von der Inanspruchnahme professioneller Hilfe überzeugen [18]. Die tertiäre Suizidprävention beschäftigt sich mit der Behandlung sowie Verbesserung der Prognose bei Personen nach einer Suizidhandlung [19].

Diesbezüglich ist anzumerken, dass unsere Ergebnisse darauf hinweisen, dass einige Kinder und Jugendliche bereits durch die ambulante Vorstellung entlastet scheinen. Trotz des häufigen Vorstellungsgrundes Suizidalität gab eine nicht unwesentliche Anzahl der Betroffenen keine oder eine geringe Ausprägung von Suizidgedanken im Erstgespräch mit der Pflege in der Akutambulanz an (75,3 % der Jungen und 58,3 % der Mädchen geben keine, kaum oder mäßige Suizidgedanken an). Ferner deutet die kurze durchschnittliche Aufenthaltsdauer im Unterbringungsbereich (durchschnittlich 2,7 Nächte) darauf hin, dass suizidale Krisen rasch beruhigt werden können. Dies sind aber nur kleine Anteile eines umfassenden Suizidpräventionsaspektes im Kindes- und Jugendalter, wobei natürlich in erster Linie wesentlich ist, Anlaufstellen außerhalb von Akutspitälern auszubauen und eine fortlaufende niederschwellige Betreuung nach der Krise anzubieten (z. B. durch niedergelassene Kinder- und Jugendpsychiater), um eine kontinuierliche Betreuung zu gewährleisten. Der Bedarf spiegelt sich unter anderem auch darin nieder, dass sich in unserer Stichprobe 22 % der Kinder und Jugendlichen erneut vorstellten und dies im Schnitt in einem relativ kurzen Zeitraum von 1–2 Monaten nach Erstvorstellung.

Unsere Ergebnisse weisen zudem darauf hin, dass für viele die Kontaktaufnahme mit der Kinder- und Jugendpsychiatrie eine Überwindung darstellt. Hier kann angenommen werden, dass durch die weiterhin bestehende Stigmatisierung von psychischen Erkrankungen eine gewisse Hemmschwelle besteht, Hilfe anzunehmen [20]. Somit ist bei psychischen Krisen häufig die übliche erste Anlaufstelle der Allgemeinmediziner, Kinder- und Jugendarzt oder die pädiatrische Akutambulanz [21]. Etwa 30 % der akut vorstelligen Patienten kamen auf Zu- oder Weiterweisung anderer ärztlicher Kollegen mit einer häufig deutlich länger bestehenden Symptomatik als bei organmedizinischen Fächern (67 % der Vorstelligen klagen über Symptome seit über 1 Woche).

Zusammenfassend ergeben sich aus unserer Studie folgende Schlussfolgerungen. Die Patienten stellen sich häufig mit einer chronifizierten Symptomatik vor. Suizidalität spielt eine große Rolle und auch eine Unterbringung zum Schutz ist häufig indiziert. Daher kann man daraus schließen, dass es sich bei akut vorstelligen Kinder und Jugendlichen um eine sehr behandlungsintensive Patientengruppe handelt.

Wir gehen davon aus, dass dieses Phänomen damit zusammenhängt, dass kinder- und jugendpsychiatrische Angebote nur mangelhaft zur Verfügung stehen und auch noch keine soziale Struktur gewachsen ist, die einen Umgang mit bestehenden Institutionen gesellschaftlich etabliert hat. Dies aber obwohl Krisen im Jugendalter häufig sind, die Suizidrate im Jugendalter im Altersvergleich die höchste ist [22, 23] und Suizide zu den häufigsten Todesursachen im Jugendalter zählen [13]. Ein Lösungsansatz könnte sein, Betroffene und Betreuungspersonen besser zu informieren, das Fortbildungsangebot im medizinischen, schulischen und psychosozialen Bereich auszuweiten [20] und gegen Stigmatisierung und Verdrängung aufzutreten sowie die kinder- und jugendpsychiatrische Betreuung im niedergelassenen Bereich auszubauen. Dadurch soll eine einfachere und bessere Inanspruchnahme einer frühzeitigen Therapie erzielt werden.

## Limitation

In anderen Regionen Österreichs gibt es womöglich andere Usancen und damit Häufigkeiten der Einweisung nach Paragraph 8 oder 9. Diesbezüglich ist uns keine Aussage möglich, da sich unsere Daten ausschließlich auf die Region Salzburg beziehen.

## Supplementary Information




