# Supplementary material for: Acute treatment of psychiatric ill children and adolescents: A pilot study on 257 patients
Source: Neuropsychiatr. 2021 Mar 15;35(4):199–205. [Article in German] doi: 10.1007/s40211-021-00389-7 (PMC8651577; doi:10.1007/s40211-021-00389-7)
Supplement: Supplementary file 1 [file 40211_2021_389_MOESM1_ESM.pdf]

UNIVERSITÄTSKLINIK FÜR KINDER- UND JUGENDPSYCHIATRIE
PMU/SALK/CDK

NF-TRIAGIERUNG

Name des Patienten: **Geburtsdatum:**

Grund der Vorstellung:

**Erstkontakt mit Pflegepersonal:**	Datum/Uhrzeit:		
**Gespräch mit Patient/in:**	ja	nein	Uhrzeit:
**Gespräch mit anderen Personen:**	ja	nein	
mit wem:			
**Bereits an der Abteilung bekannt:**	ja	nein	wann:
**Arzt/Ärztin verständigt:**	o Tagdienst	Uhrzeit:	Name:
	o Journaldienst	Uhrzeit:	Name:
**Arzt eingetroffen:**	Uhrzeit:		

Konsil: ☐ Ja ☐ nein von wo:

	Ja	Nein	falls „ja"
• Paragraph 8 oder 9?	☐	☐	=N
• Reagiert der Patient nicht altersadäquat auf Ansprache?	☐	☐	=N
• Suizidgedanken vorhanden? (wenn „Ja" suizid Skala vorlegen)	☐	☐	=N
• Fremdgefährliches Verhalten vorliegend?	☐	☐	=N
• Orientierung zu Person, Ort, Situation nicht altersadäquat vorhanden?	☐	☐	=N
• Starke Unruhe/Erregung (in Motorik oder/und innerem Erleben)	☐	☐	=N
• Fluchtgefahr	☐	☐	=N

Ab einem N = Notfall

Zur Vorstellung führende Symptome bestehen in ähnlicher Weise/Intensität seit:

RR:
Puls:

Schmerzen:

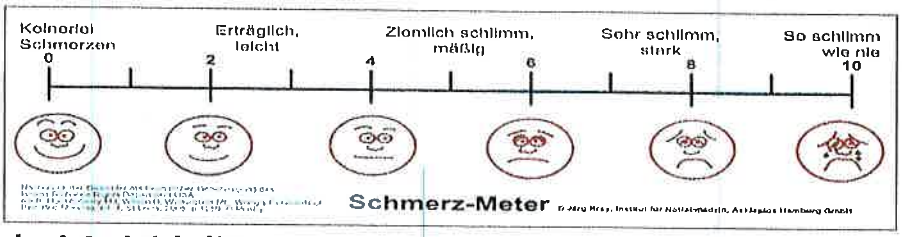

Einstufung 0-5 bei Suizidalität

0	keine	gar keine Gedanken an Suizid	gar keine Gedanken an Suizid
1	kaum	rein gedanklich	Gedanken an Suizid Schwirren rum, sind aber nicht konkret, nur ab und zu, im Sinne von Blitzgedanken, ohne emotionale Beteiligung
2	mässig	mit emotionaler Beteiligung	Gedanken sind schon etwas konkreter und öfter, verbunden mit einem gefühltem Todeswunsch
3	drängend	spürbarer Kampf	Todeswunsch und Lebenswille halten sich gerade noch die Waage, Lebensgründe sind aber noch deutlich spürbar und haben noch einen erkennbaren Wert
4	sehr drängend	Unterstützung ist absolut nötig	Todeswunsch überwiegt, die Selbstkontrolle existiert aber noch
5	Denken ist auf Suizidideen eingeschränkt	Abklärung ist zwingend nötig	d.h. ohne Unterstützung von aussen besteht keine sichere Kontrolle mehr, eine andere Person ist nötig, um wieder den notwendigen Abstand zu den Suizidgedanken zu bekommen

verweigert ☐

UNIVERSITÄTSKLINIK FÜR KINDER- UND JUGENDPSYCHIATRIE
PMU/SALK/CDK

NF-TRIAGIERUNG

Dat.		HZ

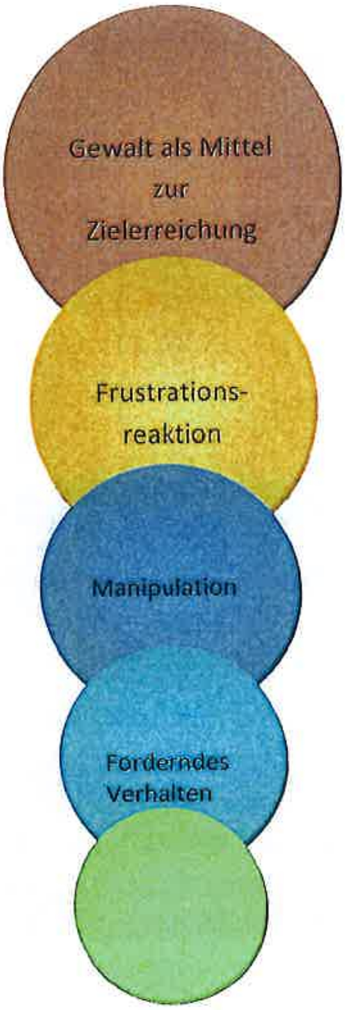

- ○ Starrer „Kalter „ Blick
  Drohende offensive Haltung
  Oft gebundene hohe Anspannung
  Sachbeschädigung

- ○ Motorische Unruhe, starke Reizbarkeit
  „Herumschimpfen" ev. Kontrollverlust
  Rascher Aufbau hoher Spannung

- ○ Erhöhter Antrieb, gezielte verbale Drohung
  Gezielte Beleidigung, mittelhohe Anspannung

- ○ Ungeduld, Reizbarkeit
  Geringe Flexibilität, hinhalten, fordernd

- ○ keine Fremdaggressionen
